# *lakemorpho*: Calculating lake morphometry metrics in R

**DOI:** 10.12688/f1000research.12512.1

**Published:** 2017-09-21

**Authors:** Jeffrey Hollister, Joseph Stachelek

**Affiliations:** 1 US Environmental Protection Agency, Office of Research and Development, National Health and Environmental Effects Research Laboratory, Atlantic Ecology Division, Narragansett, RI, USA; 2 Michigan State University, Department of Fisheries and Wildlife, Natural Resources Building, East Lansing, MI, USA

**Keywords:** limnology, R, lake morphometry, lake depth, lake volume

## Abstract

Metrics describing the shape and size of lakes, known as lake morphometry metrics, are important for any limnological study. In cases where a lake has long been the subject of study these data are often already collected and are openly available. Many other lakes have these data collected, but access is challenging as it is often stored on individual computers (or worse, in filing cabinets) and is available only to the primary investigators. The vast majority of lakes fall into a third category in which the data are not available. This makes broad scale modelling of lake ecology a challenge as some of the key information about in-lake processes are unavailable. While this valuable *in situ* information may be difficult to obtain, several national datasets exist that may be used to model and estimate lake morphometry. In particular, digital elevation models and hydrography have been shown to be predictive of several lake morphometry metrics. The R package *lakemorpho *has been developed to utilize these data and estimate the following morphometry metrics: surface area, shoreline length, major axis length, minor axis length, major and minor axis length ratio, shoreline development, maximum depth, mean depth, volume, maximum lake length, mean lake width, maximum lake width, and fetch. In this software tool article we describe the motivation behind developing *lakemorpho*, discuss the implementation in R, and describe the use of *lakemorpho* with an example of a typical use case.

## Introduction

The study and quantification of lake shape (i.e. lake morphology and morphometry) is one of the foundations of limnology, and for students of limnology, some of the first lessons are centered around a typical suite of metrics and how to calculate them ^1^. It is also widely accepted that the morphometry of lakes and ponds can impact available nutrients and thus overall productivity. For instance, the widely used Vollenweider input-output models that are used to estimate nutrient concentrations rely on hydraulic residence time and sometimes mean depth, both of which are derived from total lake volume ^2, 3^. Also, clear water versus turbid water states in lakes have been linked in part to lake morphometry, in particular mean depth ^4, 5^. In short, limnologists have long recognized the importance of lake morphology as one factor controlling a variety of ecological processes in lakes.

Traditional methods for calculating lake morphometry metrics have relied upon the use of paper bathymetry maps, planimeters, or simple heuristics ^6– 9^. In addition, detailed bathymetry is a requirement for the calculation of most lake morphometry metrics, but is generally only available for a relatively small number of lakes. Although this is not a problem when the focus of a study is a single lake, a small number of lakes, or a group of well-studied lakes, reliance on complete bathymetry becomes a limitation when attempting to conduct regional or national-scale lake studies. For instance, Soranno *et al.* found that for some water quality datasets lake depth, in spite of its importance, was not always available ^10^. In cases such as these, alternative approaches for estimating lake morphometry are required.

Recent work has demonstrated the ability to estimate many of these metrics from ubiquitous spatial data ^9, 11– 13^. For instance, maximum depth and lake volume may be predicted using the lake polygon and surrounding topography ^9, 11^ provided by the National Hydrography Dataset Plus and the National Elevation Dataset, respectively ^14, 15^. These methods were initially developed with proprietary tools, thus limiting their accessibility. In an effort to reach a broader audience, the tools were converted to R, expanded to include a more complete suite of lake morphometry metrics and compiled into an R Package.

## Methods

### Implementation in R

Using R as a Geographic Information System is now possible, as several packages provide spatial data handling, geospatial analysis, and visualization. It is because of these packages that lakemorpho was implemented as an R package ^16^. In particular, lakemorpho relies on the following packages: maptools, rgdal, raster, rgeos, sp, and geosphere ^17– 23^. In addition to these packages, two external libraries, the Geospatial Data Abstraction Library (GDAL) and Geometry Engine, Open Source (GEOS), are needed. Their availability to R and installation varies by operating system ^24, 25^.

### Using lakemorpho

The lakemorpho package includes one function to create a lakeMorpho object, several functions to calculate morphometry metrics, a default plotting function, two example datasets, and an example lakeMorpho object.

A typical workflow for using lakemorpho to calculate lake metrics would include pulling spatial data into R (e.g. as shapefiles, tiff, etc.), creating a lakeMorpho object and calculating the desired lake morphometry metrics. The following sections provide details on the type of input data required and demonstrate use of the available functions with the provided example data.

**Figure 1.**
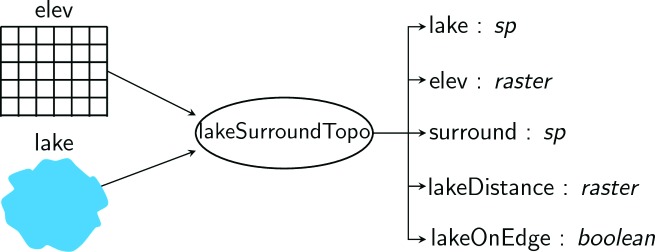
Diagram of lakemorpho object.

***The lakeMorpho Class and lakeSurroundTopo function.*** Many of the lake morphometry metrics rely on the same information about the lake. For instance, the functions to estimate maximum depth, mean depth, and volume rely on statistical summaries of the surrounding topography as well as the maximum in-lake distance to shoreline ^9, 11^. To avoid recalculating these values, a lakeMorpho object was created to link information on surrounding topography to the original datasets and facilitate default plotting of the outputs. All lake morphometry functions in the lakemorpho package require an object of class lakeMorpho as input. Some functions also return an updated lakeMorpho object that includes calculated spatial objects as output. At a minimum, a lakeMorpho object contains (see Figure 1):

- "lake" - A SpatialPolygons or SpatialPolygonsDataFrame object of the original input lake data.
- "elev" - A RasterLayer representing the elevation in a suitably large area around the lake.
- "surround" - A SpatialPolygons or SpatialPolygonsDataFrame object representing the land area defined as the surrounding topography.
- "lakeDistance" - A RasterLayer object of the euclidean distance from the shoreline to center of each pixel. Maximum value is equal to the maximum in-lake distance.
- "lakeOnEdge" - A logical value indicating if the "surround" polygon falls on the edge of the "elev" raster (i.e. would contain missing (i.e. NA) elevation data).

The lakeSurroundTopo function is the primary mechanism for creating a lakeMorpho object. There are two required inputs and one optional input for lakeSurroundTopo. The first required input is a SpatialPolygons or SpatialPolygonsDataFrame of the lake ^21^. Only a single lake is accepted as input, although this lake may be composed of multiple polygons (i.e. a lake with islands). If metrics for multiple lakes are required they will need to be passed to the suite of lakemorpho functions separately. The second required input is a RasterLayer of the elevation surrounding the lake ^22^. The default raster size is taken from the resolution of the input elevation data but may be specified separately. The third input specifies the area representing the surrounding topography. By default, this is a buffer of the lake shoreline, with the buffer width equal to the maximum in-lake distance. An optional SpatialPolygons object of any polygon intersecting the lake (e.g. catchments) can be used to define the surrounding topography instead of the default buffer. An object of class l akeMorpho is returned from lakeSurroundTopo.

In addition to providing the required inputs, users should pay attention to both the extent of the input elevation dataset as well as the coordinate reference systems being used. First, the elevation data must be of a large enough extent so that the surrounding topography does not include land area outside that extent (i.e. would return NA values). As noted above, the lakeOnEdge item indicates if the surrounding topography is on the edge of the user supplied elevation and thus would return some missing data. Second, all of the functions of lakemorpho assume that projections have been handled prior to creating the lakemorpho class or calculating the metrics. If the input data are not of the same projection, lakeSurroundTopo will return an error. The data must be re-projected into the same coordinate reference system (CRS). The units of all metrics are determined by the CRS and care must be taken to make sure that the vertical units of the elevation are the same as horizontal units of the projection. For instance, elevation data may be available in meters yet the CRS is specified in feet. In cases such as these, a conversion of the vertical data should be done. Lastly, care must be taken in choosing an appropriate CRS for the area under consideration. This is because all CRS will distort area, distance, shape, or direction. Thus a projection that minimizies distortions of distance and area are preferrable. A useful reference for further exploring coordinate reference system is Iliffe and Lott's 2008 book on the topic ^26^.

Usage of lakeSurroundTopo and generation of a lakeMorpho object from the example data included with lakemorpho is done as follows:



                        #Load data

                        data
                        (lakes)


                        #Create lakeMorpho object, example_lakeMorpho, with required inputs

                        example_lakeMorpho <- 
                        lakeSurroundTopo
                        (exampleLake, exampleElev)
                    


The resulting object contains the minimum set of components that make up a lakeMorpho object. We can verify that the components are of the expected class with the following command:



                        lapply
                        (example_lakeMorpho,class)

## $lake
## [1] "SpatialPolygonsDataFrame"
## attr(,"package")
## [1] "sp"
## 
## $elev
## [1] "RasterLayer"
## attr(,"package")
## [1] "raster"
## 
## $surround
## [1] "SpatialPolygons"
## attr(,"package")
## [1] "sp"
## 
## $lakeDistance
## [1] "RasterLayer"
## attr(,"package")
## [1] "raster"
## 
## $lakeOnEdge
## [1] "logical"

                    


***Lake Morphometry Functions.*** Each of the remaining functions expects a lakeMorpho object as input and returns a numeric value. Some of the functions also have a side effect of adding a spatial object to the input lakeMorpho object.

**calcLakeMetrics**

The calcLakeMetrics function is a convenience function that will calculate all of the lakemorpho metrics for a single lakeMorpho object. It requires an input lakeMorpho object, a bearing for calculating lakeFetch, and pointDens for maximum lake length and width (defined below).



                        calcLakeMetrics
                        (example_lakeMorpho, 
                        0
                        , 
                        250
                        )

## $surfaceArea
## [1] 16453180
## 
## $shorelineLength
## [1] 45991.38
## 
## $shorelineDevelopment
## [1] 3.198502
## 
## $maxDepth
## [1] 99.17621
## 
## $volume
## [1] 4802535
## 
## $meanDepth
## [1] 28.94864
## 
## $maxLength
## [1] 9479.313
## 
## $maxWidth
## [1] 3166.225
## 
## $meanWidth
## [1] 1735.693
## 
## $fetch
## [1] 6336.798

                    


**lakeFetch**

Fetch is the maximum open water distance in a given direction and can be used an indicator of mixing as greater fetch implies greater potential for waves due to wind effects ^27^. The lakeFetch function calculates fetch along an input bearing. The input bearing may be any value from 0 to 360 where 0 and 360 both represent north, and the fetch for opposite directions (e.g. east and west) are identical.

To calculate the fetch of an input lake use:



                        #Fetch for north

                        lakeFetch
                        (example_lakeMorpho, 
                        0
                        )


                        ## [1] 6336.798


                        lakeFetch
                        (example_lakeMorpho, 
                        360
                        )


                        ## [1] 6336.798


                        #Fetch for west

                        lakeFetch
                        (example_lakeMorpho, 
                        270
                        )


                        ## [1] 3129.997
                    


**lakeMajorAxisLength**

The major axis of a lake is defined as the longest line intersecting the convex hull formed around its polygon while passing through its center. In contrast to lakeMaxLength, its value represents the distance across a lake without regard to land-water configuration.

To calculate the major axis length of an input lake use:



                        lakeMajorAxisLength
                        (example_lakeMorpho, 
                        addLine = 
                        TRUE
                        )


                        ## [1] 13159.64
                    


**lakeMaxDepth**

Maximum lake depth provides information that may be used, along with flow rates, to estimate the residence time of a lake. While there is no substitute for field verified measurements, maximum lake depth may be estimated from the surrounding topography. The lakeMaxDepth function uses the methods outlined in Hollister *et al.* ^11^ to provide an estimate of the maximum lake depth. It requires only a lakeMorpho object as input. Optionally, a correction factor based off of verified depth data may be specified if one is known.

To calculate maximum depth use:



                        #Maximum lake depth

                        lakeMaxDepth
                        (example_lakeMorpho)

## [1] 99.17621
                    


It is important to note that the accuracies of these maximum depth predictions do vary across regions and often a correction factor using field measured data is required. For example, Hollister *et al.* ^11^ demonstrate that for the New England and Mid-Atlantic regions of the United States East coast, the initial predictions were larger than the true values and needed to be reduced.

**lakeMaxLength**

**Figure 2.**
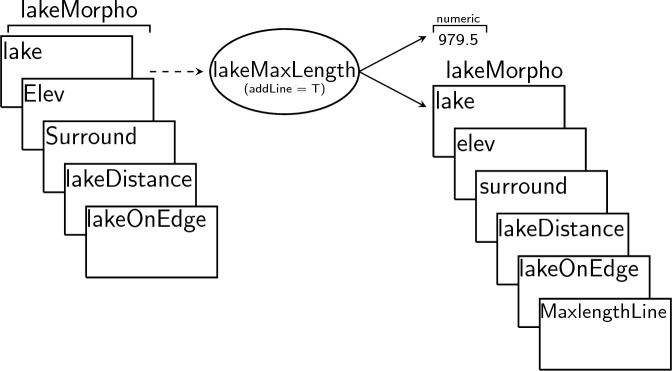
Diagram of the lakemorpho object before and after calculating a lake metric.

Maximum lake length is the longest open water distance within a lake and, similar to fetch, is a metric that can be used to estimate mixing potential ^1, 28^. The current implementation of this calculation in lakemorpho places points at equal distances along the shoreline of the lake and then finds the longest point-to-point distance that also does not intersect land (e.g. peninsulas or islands). The optional parameter addLine has a default value of TRUE and allows the SpatialLines object to be stored on the input lakeMorpho object ( Figure 2).

To calculate maximum lake length use:



                        #Max length with a point density of 250

                        lakeMaxLength
                        (example_lakeMorpho, 
                        250
                        , 
                        addLine = 
                        FALSE
                        )


                        ## [1] 9025.769
                    


The pointDens parameter can have an impact on both the processing time and the resulting value and both of these can vary as a function of the complexity of the shape of the lake with less complex lakes providing more consistent lake length across a number of points. Given this caveat, care must be taken in choosing an appropriate number of points (and thus lines) to use to calculate maximum lake length. Several densities should be tested and the smallest number of points that produce a stable estimate should be used.

**lakeMaxWidth**

Maximum lake width is the maximum shore to shore distance that is perpendicular to the line representing maximum lake length and is another metric related to mixing ^1, 28^. The lakeMaxWidth function requires a lakeMorpho object and pointDens value which is used to determine the number of points along the maximum lake length line. The issue with pointDens, discussed above, also exists for the use of pointDens with lakeMaxWidth and care should be taken to determine an appropriate number of lines to test.

Usage of lakeMaxWidth is:



                        #Max width with a point density of 250

                        lakeMaxWidth
                        (example_lakeMorpho, 
                        250
                        )


                        ## [1] 3177.625
                    


**lakeMeanDepth**

Mean depth of a lake is calculated as the volume of the lake divided by the area ^1, 28^. This function requires only a lakeMorpho object and returns a numeric value of the mean depth. Usage of the function is:



                        lakeMeanDepth
                        (example_lakeMorpho)


                        ## [1] 28.94864
                    


There is an optional zmax argument that allows a user to specify a maximum lake depth if one is available. If not supplied, the maximum depth will be estimated using lakeMaxDepth. For instance, in the above example, the maximum depth without using a correction factor is estimated at 99 meters which results in a mean depth estimate of 28.95. The measured maximum depth, 32 meters, is much less than the estimate depth. To use this information you would simply add the measured valued in for the zmax argument.



                        lakeMeanDepth
                        (inputLM, 
                        zmax = 
                        32
                        )


                        ## [1] 9.340511



**lakeMeanWidth**

The mean width of a lake is defined as lake area divided by maximum lake length ^1, 28^. Input for this function is a lakeMorpho object that has the maximum lake length line added via 'lakeMaxLength`. This requirement is checked and returns an error if the maximum length line is missing.



                        # Add the maximum lake length line

                        lakeMaxLength
                        (example_lakeMorpho, 
                        pointDens = 
                        100
                        , 
                        addLine = 
                        TRUE
                        )


                        ## [1] 8194.247


                        # Calculate mean width

                        lakeMeanWidth
                        (example_lakeMorpho)


                        ## [1] 2007.894



**lakeMinorAxisLength**

The minor axis of a lake is defined as the shortest line intersecting the convex hull formed around the lake polygon while passing through its center. In contrast to lakeMaxWidth, its value represents the distance across a lake with regard to the the convex hull and without consideration of the land-water configuration.



                        lakeMinorAxisLength
                        (example_lakeMorpho, 
                        addLine = 
                        TRUE
                        )


                        ## [1] 6926.263



**lakeMinorMajorRatio**

The ratio of the lake major axis length to the minor axis length is also known as the aspect ratio. Circular lakes have aspect ratios approaching 1 while thin-elongated lakes have aspect ratios approaching 0. If major and minor axis length have not already been added to the lakeMorpho object, these are calculated. The addLine argument adds the lines for the lake's minor and major axes to the lakeMorpho object.



                        lakeMinorMajorRatio
                        (example_lakeMorpho, 
                        addLine = 
                        TRUE
                        )


                        ## [1] 0.5263261



**lakeShorelineDevelopment**

The shoreline development metric provides a measure of the complexity of the shoreline. It is a ratio of the perimeter of the lake to the perimeter of a circle of the same area. Values will be 1 or greater with value of 1 indicating a circular lake. This metric is used as an indicator of potential habitat ^1, 28^. It only requires a lakeMorpho object as input.



                        lakeShorelineDevelopment
                        (example_lakeMorpho)


                        ## [1] 3.198502



**lakeShorelineLength and lakeSurfaceArea**

Shoreline length is simply the total perimeter of the lake polygon and, as with all other functions, requires a lakeMorpho object as input. To calculate the shoreline length:



                        lakeShorelineLength
                        (example_lakeMorpho)


                        ## [1] 45991.38



Similarly, surface area for a lake is the total area of the lake polygon. It is calculated via:



                        lakeSurfaceArea
                        (example_lakeMorpho)


                        ## [1] 16453180



**lakeVolume**

The lakeVolume function uses maximum lake depth (see lakeMaxDepth) and methods outlined by Hollister *et al.* ^9^ to estimate lake volume. The method assumes that the maximum in-lake distance (D _max_) from the shoreline is also the deepest part of the lake (Z _max_). The lakeVolume function creates a raster of the in-lake distance to shoreline and converts those distances, using Z _max_:D _max_, to depths and then summing the volume of each pixel to estimate total lake volume.



                        lakeVolume
                        (example_lakeMorpho)


                        ## [1] 476297184
                    


Similar to lakeMeanDepth, there is a zmax argument to be used for a known maximum lake depth.

## Use case

**Figure 3.**
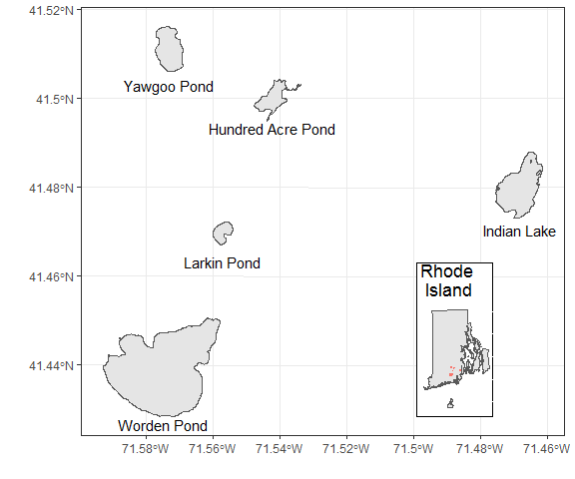
Map of lakes used in the case study example of the lakemorpho package.

A common application of lakemorpho is to calculate the full suite of lake metrics for multiple lakes. This use case demonstrates how to do that with a commonly encountered GIS data file, the shapefile. To do this we iterate through the lakes, calculate metrics for each lake and include the metrics on an output shapefile. The data for this use case is from Rhode Island ( Figure 3). The data for the lakes were downloaded from the Rhode Island Geographic Information Systems (RIGIS) ^29^ and the elevation data are from Amazon Web Services Terrain Tiles via the elevatr package ^30^.

### Read in data

This use case relies on the sp and rgdal packages for the spatial data handling. These are dependencies for lakemorpho, thus no additional installs are required. To read in the data we utilized rgdal::readOGR and read in the ri_lakes.shp from the current directory. This file is available for download from https://github.com/USEPA/lakemorpho_manuscript/blob/master/ri_lakes.zip.



                        # Load packages

                        library
                        (
                        "sp"
                        )

                        library
                        (
                        "rgdal"
                        )

                        library
                        (
                        "lakemorpho"
                        )
                    




                        # Read the shapefile and plot

                        ri_lakes <- 
                        readOGR(
                        "."
                        ,
                        "ri_lakes"
                        )


                        ## OGR data source with driver: ESRI Shapefile 
## Source: ".", layer: "ri_lakes"
## with 5 features
## It has 2 fields
                    


### Iterate through lakes and calculate metrics

In R, there are many ways to iterate. For simplicity and clarity we use a for loop to iterate through all lakes and calculate the full suite of lake metrics with calcLakeMetrics. We will utilize the elevatr package which provides access to elevation data from various sources ^30^. In this example we will use the Amazon Web Services terrain tiles. The vertical elevation data are in meters and the Rhode Island lake data are projected in Rhode Island State Plane Feet, thus we will convert the elevation data into feet.



                        library
                        (elevatr)

                        output <- 
                        data.frame
                        ()

                        for
                        (i 
                        in seq_along
                        (ri_lakes)){

                          dem <- 
                        get_elev_raster
                        (ri_lakes[i,],
                        z = 
                        12
                        , 
                        expand = 
                        1000
                        , 
                        src = 
                        "aws"
                        ) 
                        * 
                        3.281

                          lmorph <- 
                        lakeSurroundTopo
                        (
                        inLake = 
                        ri_lakes[i,], 
                        inElev = 
                        dem)

                          lmetric <- 
                        calcLakeMetrics
                        (lmorph, 
                        bearing =
                         270
                        , 
                        pointDens = 
                        100
                        )

                          output <- 
                        rbind
                        (output,

                                          data.frame
                        (
                        NAME = 
                        ri_lakes[i,]
                        $
                        NAME,

                                                     data.frame
                        (lmetric)))

                        }
                    


We can now merge the morphometry metrics back to the lake polygons.



                        ri_lakes_m <- 
                        merge
                        (ri_lakes,output,
                        by=
                        "NAME")

                        dplyr::tbl_df
                        (ri_lakes_m)


                        ## # A tibble: 5 x 12
##                NAME   Acres surfaceArea shorelineLength
## *            <fctr>   <dbl>       <dbl>           <dbl>
## 1       Indian Lake  268.55    11698076        18857.94
## 2       Yawgoo Pond  144.37     6288693        10562.24
## 3       Worden Pond 1098.64    47856596        34494.32
## 4       Larkin Pond   43.85     1910010         5989.30
## 5 Hundred Acre Pond   87.64     3817524        15278.75
## # ... with 8 more variables: shorelineDevelopment <dbl>, maxDepth <dbl>,
## #   volume <dbl>, meanDepth <dbl>, maxLength <dbl>, maxWidth <dbl>,
## #   meanWidth <dbl>, fetch <dbl>
                    


## Conclusions

The lakemorpho package provides functions to calculate common lake morphometry metrics in R. For those conducting lake analyses in R this allows for streamlined analysis workflows. Also, lakemorpho provides a foundation for additional metrics. For instance, it might be possible to combine hydrological methods for estimating stream flow into and out of lakes with lake volume and add a function for calculating residence time.

Beyond adding additional metrics, more fundamental rewriting of the package may also be useful. For instance, lakemorpho currently is built on top of the current spatial data standard for R, sp. This allows a clean interface with many existing tools; however, it is likely that sp will be replaced in the next several years by the sf package ^21, 31^. Future versions of lakemorpho might benefit from using the sf tool chain and the "tidy data" framework ^32^.

In summary, lakemorpho provides limnologists and aquatic ecologists with a consistent framework in R for calculating a suite of the most common lake morphometry metrics. This paper outlines the currently available functions and provides an example through a typical use case of calculating many metrics for several lakes.

## Software availability

The lakemorpho version 1.1.0 package is currently available directly from the Comprehensive R Archive Network (CRAN) and may simply be installed and loaded in R via:



                    install.packages
                    (
                    'lakemorpho'
                    )

                    library
                    (
                    'lakemorpho'
                    )
                


To access the help pages (including a version of this manuscript) use:



                    help
                    (
                    package=
                    'lakemorpho'
                    )
                


There are tentative plans to continue developing new functions for lakemorpho and these new features will be available first through the development version on GitHub at http://github.com/usepa/lakemorpho.

To install and load the development version requires use of the devtools package. This may be done with:



                    install.packages
                    (
                    'devtools'
                    )

                    library
                    (
                    'devtools'
                    )

                    install_github
                    (
                    'USEPA/lakemorpho'
                    )

                    library
                    (lakemorpho)
                


Archived source code as at the time of publication:

http://doi.org/10.5281/zenodo.863051 ^33^
