# Supplementary material for: lakemorpho: Calculating lake morphometry metrics in R
Source: F1000Res. 2017 Sep 21;6:1718. [Version 1] doi: 10.12688/f1000research.12512.1 (PMC5698920; doi:10.12688/f1000research.12512.1)
Supplement: Peer review history

# Peer review: Referee response for version 1

**DOI:** 10.5256/f1000research.13548.r27253

**Published:** 2017-11-20

**Authors:** Luke A. Winslow

**Affiliations:** 1 Rensselaer Polytechnic Institute, Troy, NY, USA

In this manuscript, the Hollister and Stachelek outline the functionality and use of the lakemorpho R package. The package includes a number of tools that allow users to calculate morphological, including a some generally complex metrics that are not easy to recreate using existing functionality in available spatial packages. Packages such as these go a long way to preventing the constant re-inventing of the wheel by graduate students and other researchers in generating code to calculate these metrics. This package is a significant contribution to the field and this paper does a reasonable job of describing the package.

 I have a few minor suggestions that I think will improve the manuscript and applicability of the package.

 Some of these metrics are not immediately intuitive to most readers. It would be super useful to have a figure, maybe with a series of panels, that shows the reader what each of these metrics are on some example lakes where it matters. This would be great to have accompanying the section “Lake Morphology Functions”.

 There needs to be a description of what the option ‘pointDens’ means. Does it have units? Is it relative to the projection or independent of it? Further, while this may be difficult, would it be possible to show the sensitivity of output metrics to pointDens for complex lakes? It would be great to know if there was a reliable metric that could be used, perhaps a default informed by the total lake area or SDF? Such a default or heuristic would be really useful to the purported use here (calculations on a large number of less-studied lakes). Basically, if I use this on 1 million lakes at the continental scale, I really need an informed way to pick that pointDens value.

 Right now, this package has depth-related calculations for lakes which do not have observed bathymetry. It would be really useful if the package could be generalized to use observed bathymetry rasters in the calculation of max and mean depth. Such data are increasingly available (e.g. http://bathybase.org).

 A lot of 1-D hydrodynamic models need an estimate of the hypsometric curve. It would be great if an additional function could be included that would use either an estimated bathymetry or a provided bathy raster (see earlier comment) and output the lake’s hypsometric curve. Very useful in hydrodynamic modeling.  

 Here are a few additional publications you might consider referencing. At the moment, references to existing lake morphological literature is a little thin. Especially check out other work of Håkanson as he’s a morphology master.

 Håkanson, L. (2005), The Importance of Lake Morphometry for the Structure and Function of Lakes ^1^

 Dunalska, J. a. (2011), Impact of morphometric and catchment variables on summer organic carbon richness in deep temperate lakes ^2^

 Read, E. K. et al. (2015), The importance of lake-specific characteristics for water quality across the continental United States ^3^

 I look forward to using this useful tool!

I have read this submission. I believe that I have an appropriate level of expertise to confirm that it is of an acceptable scientific standard.

## References

[1] HåkansonL: The Importance of Lake Morphometry for the Structureand Function of Lakes. *International Review of Hydrobiology*.2005;90(4) : 10.1002/iroh.200410775 433-461 10.1002/iroh.200410775

[2] DunalskaJ: Impact of morphometric and catchment variables on summer organic carbon richness in deep temperate lakes. *Knowledge and Management of Aquatic Ecosystems*.2011; 10.1051/kmae/2011043 10.1051/kmae/2011043

[3] ReadEPatilVOliverSHetheringtonABrentrupJZwartJWintersKCormanJNodineEWoolwayRDuganHJaimesASantosoAHongGWinslowLHansonPWeathersK: The importance of lake-specific characteristics for water quality across the continental United States. *Ecological Applications*.2015;25(4) : 10.1890/14-0935.1 943-955 10.1890/14-0935.1 26465035

# Peer review: Referee response for version 1

**DOI:** 10.5256/f1000research.13548.r26223

**Published:** 2017-10-23

**Authors:** Samantha K. Oliver

**Affiliations:** 1 Center for Limnology, University of Wisconsin-Madison, Madison, WI, USA

The *lakemorpho *package fills a need in the limnological community (and beyond) for easily deriving lake morphometry metrics in a repeatable and documented way. The package is relatively simple to use and understand, and implements common, published methods for calculate lake morphometry metrics. Additionally, the authors implement a tool for predicting lake depth, which is a difficult lake parameter to obtain. I only a have minor suggestions for changes in the language and documentation for the package. Most of these changes are related to the order in which ideas are presented in the paper. While reading the paper, I had several questions that were later answered, but I think the authors can avoid the questions altogether by simply rearranging some of the text (see specific comments below). Additionally, I think it’s important for the authors to include an error estimate of the lake depth predictions – prediction error is notoriously high for lake depth, and I think it’s important for users to understand this. I recommend this paper for publication after the following changes are considered – and I look forward to using the package!

 Minor revisions:

 - Recommend removing “…and for students of limnology, some of the first lessons are centered around a typical suite of metrics and how to calculate them.” from the first sentence and instead lead with morphometry affecting water quality.

 - What is a “suitably large area around the lake”? 50m, 100m?

 - I think you can remove bulleted list from the first paragraph of the *lakeMorpho* and *lakeSurroundTopo* section. It only roughly introduces pieces of the *lakeMorpho* object, and left me with more questions than answers (e.g., previous question RE: suitably large area around the lake which you answer later). You answered all of the questions immediately in the next paragraphs, which left me feeling like you could just leave out the list, or use it as a summary list at the end of the section.

 - “If metrics for multiple lakes are required they will need to be passed to the suite of lakemorpho functions separately.” This seems to be in direct contradiction with the purpose of the package – which is to derive morphometry metrics for a large number of lakes. It

 becomes clear later in the text that you address this with a use-case. I think you should state this up front – “Although each lake must be passed through lakemorpho functions separately, we provide a use case to show how a user can easily calculate these metrics for several lakes”.

 - This sentence is really confusing: “First, the elevation data must be of a large enough extent so that the surrounding topography does not include land area outside that extent (i.e. would return NA values)”. My interpretation is: “The extent of the elevation data must be equal to or greater than the extent of the area surrounding the lake that you are summarizing”. I think this is confusing because you are not using “surrounding topography” in a general sense but as a defined area that is part of the user input. Perhaps use a different naming convention? You’re describing the surrounding topography (through summarizing the elevation data) within a specified area. Perhaps elevation = your fabric and buffer area = your stencil? This is adopting language from the USGS geoknife package. It may not make sense to use fabric since you only have one type of raster input, but I think stencil is a nice word to use for your variable buffer/watershed area. I see you call this “surround” in the lakemorpho object, so that may be a good option as well.

 - Maybe give some error measurement from the max depth predictions in the Hollister paper? I think it’s important for users to understand there is substantial error in these models.

 - I think you should briefly describe the Hollister methods for calculating zmax in this paper. Also, the mean depth calculation is unclear from the text. How is volume calculated? By estimating depth at each pixel of the lake? Do you know anything about the error of these estimates? Ok – now I see that you give a good description of volume. Maybe move volume up earlier (before mean depth)?

 - In the lake shoreline development section: “This metric is used as an indicator of potential habitat” It is also used an indicator of potential terrestrial-aquatic linkages ^1^.

I have read this submission. I believe that I have an appropriate level of expertise to confirm that it is of an acceptable scientific standard.

## References

[1] WinslowLReadJHansonPStanleyE: Lake shoreline in the contiguous United States: quantity, distribution and sensitivity to observation resolution. *Freshwater Biology*.2014;59(2) : 10.1111/fwb.12258 213-223 10.1111/fwb.12258
